# Choroidal Neovascularization: Mechanisms of Endothelial Dysfunction

**DOI:** 10.3389/fphar.2019.01363

**Published:** 2019-11-29

**Authors:** Natalie Jia Ying Yeo, Ebenezer Jia Jun Chan, Christine Cheung

**Affiliations:** ^1^Lee Kong Chian School of Medicine, Nanyang Technological University, Singapore, Singapore; ^2^Division of Psychology, School of Social Sciences, College of Humanities, Arts, and Social Sciences, Nanyang Technological University, Singapore, Singapore; ^3^Duke-NUS Medical School, National University of Singapore, Singapore, Singapore; ^4^Institute of Molecular and Cell Biology, Agency for Science, Technology and Research, Singapore, Singapore

**Keywords:** choroidal neovascularization, endothelial, vascular mechanisms, age-related macular degeneration, disease models

## Abstract

Many conditions affecting the heart, brain, and even the eyes have their origins in blood vessel pathology, underscoring the role of vascular regulation. In age-related macular degeneration (AMD), there is excessive growth of abnormal blood vessels in the eye (choroidal neovascularization), eventually leading to vision loss due to detachment of retinal pigmented epithelium. As the advanced stage of this disease involves loss of retinal pigmented epithelium, much less attention has been given to early vascular events such as endothelial dysfunction. Although current gold standard therapy using inhibitors of vascular endothelial growth factor (VEGF) have achieved initial successes, some drawbacks include the lack of long-term restoration of visual acuity, as well as a subset of the patients being refractory to existing treatment, alluding us and others to hypothesize upon VEGF-independent mechanisms. Against this backdrop, we present here a nonexhaustive review on the vascular underpinnings of AMD, implications with genetic and systemic factors, experimental models for studying choroidal neovascularization, and interestingly, on both endothelial-centric pathways and noncell autonomous mechanisms. We hope to shed light on future research directions in improving vascular function in ocular disorders.

## Introduction

Endothelial dysfunction underlies the crux of many conditions, which may implicate comorbidities. One example is choroidal neovascularization, a process in wet or exudative age-related macular degeneration (AMD), characterized by the abnormal intravasation of choroidal vasculature into the retinal epithelium or subretinal tissue. This often involves dysfunctional and leaky vessels, which then lead to the accumulation of fluid and blood in the macula (Chirco et al., 2017; Saini et al., 2017). AMD is the principal cause of permanent blindness among elderly over 60 years in industrialized countries (Stan et al., 2004; Pascolini and Mariotti 2012; Wong et al., 2014). It has a prevalence of 8.7% which will increase with ageing populations, adversely affecting the quality of life of 196 million people by 2020. As one would expect, it will incur substantial public health burden in the next few decades (Friedman et al., 2004; Seddon et al., 2005; Wong et al., 2014; Jonas et al., 2017). Among AMD cases with acute visual impairment, wet AMD is responsible for approximately 90% of cases (Ferris et al., 1984). Despite the rising prevalence of this debilitating condition, current treatment strategies for wet AMD mostly revolve around inhibitors of vascular endothelial growth factor and photodynamic therapy. Both have considerable limitations such as lack of long-term improvement on visual acuity (Rofagha et al., 2013; Fernández-Robredo et al., 2014; Bracha et al., 2017; Dunn et al., 2017; Jaffe et al., 2017; Malek et al., 2018) and secondary inflammatory side effects (Ho et al., 2016). These bring to light the necessity for a deeper understanding of the disease (Fernández-Robredo et al., 2014; Bracha et al., 2017; Malek et al., 2018).

Endothelial dysfunction plays a role in many human diseases. Patients with early vascular abnormalities have been found to acquire AMD and associated cardiovascular and cerebrovascular diseases later in their lives (Cheung and Wong, 2014). Research on AMD has mainly focused on retinal pigmented epithelium deficit as that is the ultimate pathological change leading to vision loss, whereas mechanisms of endothelial dysfunction in choroidal neovascularization remain elusive. Limitations with the current gold standard treatment for wet AMD using inhibitors of vascular endothelial growth factor (VEGF) have revealed possibilities of VEGF-independent pathways (Huang et al., 2016). Despite advances in genome wide association studies (GWAS), risk variants associated with AMD are hardly translated into the intended development of diagnostics and treatment. It is slowly being recognized that genetic risk variants exert minuscule influences as they often have no direct relevance to the illness. In fact, they are postulated to act through complex regulatory networks to influence the activity of key genes that are more biologically connected to the disease (Boyle et al., 2017).

We recognize that emerging studies are discovering a significant involvement of endothelial pathology in choroidal neovascularization. Here, we provide a nonexhaustive review to address the vascular underpinnings of AMD, provide information on state-of-the-art experimental models of choroidal neovascularization, and interpret existing knowledge on endothelial mechanisms with heterotypic interplay of different cell types and environmental factors.

## Vascular Etiology in Age-Related Macular Degeneration

### Endothelial Dysfunction in Early Stages

**Figure 1:**
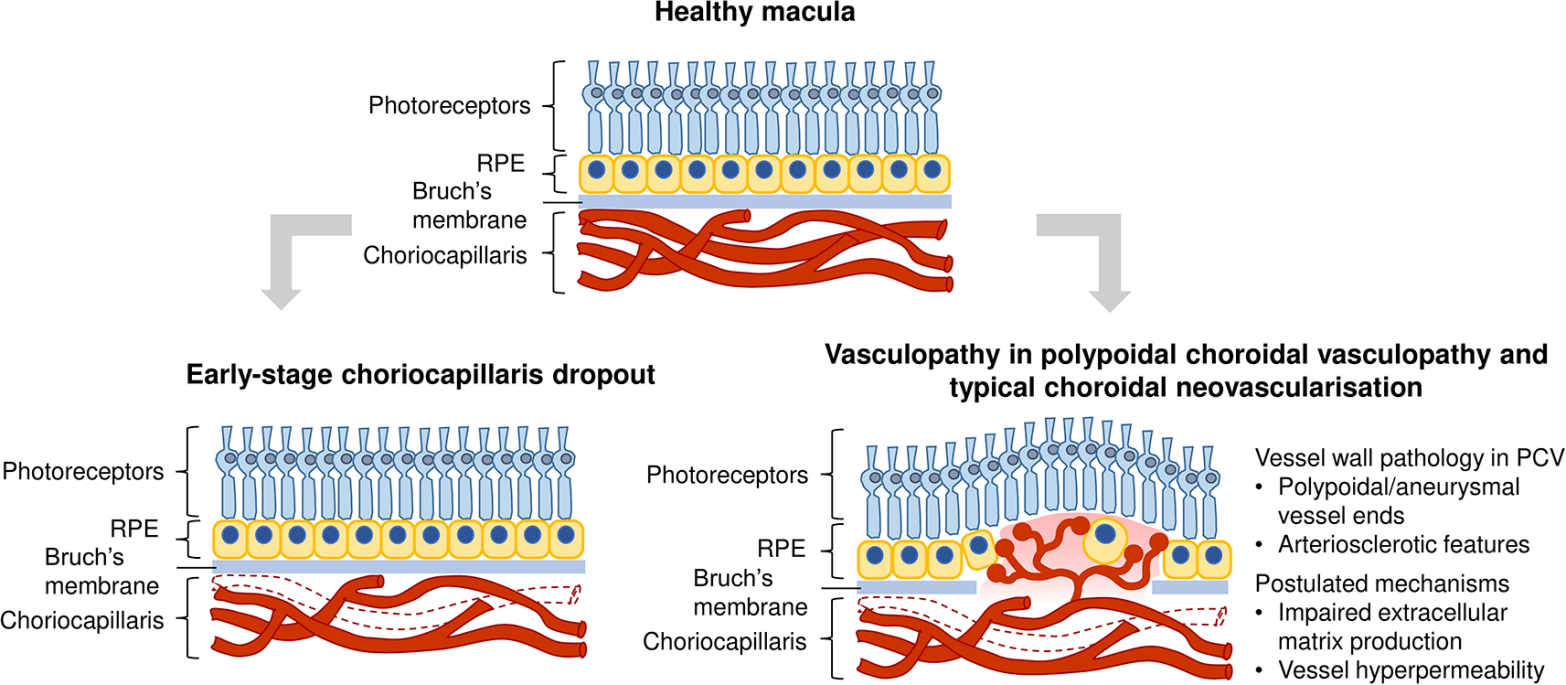
Vascular contribution to wet age-related macular degeneration (AMD) pathogenesis. Top image: Healthy macula; Bottom left image: Vascular changes could occur early in disease progression, manifested as a reduction in choriocapillaris density through loss of endothelial cells; Bottom right image: Vasculopathy is also observed in polypoidal choroidal vasculopathy and typical choroidal neovascularization, such as polypoidal/aneurysmal dilations of vessels and arteriosclerotic features. Mechanisms in common vessel wall pathology in polypoidal choroidal vasculopathy (PCV) include impaired extracellular matrix production and choroidal vascular hyperpermeability.

While the pathogenesis of wet AMD is still poorly understood, several reports suggest a vascular etiology for the disease. The choroidal endothelial cells that form choriocapillaris vessel walls are lost even before the occurrence of retinal pigmented epithelium dysfunction, suggesting that vascular dysfunction could be the first trigger of wet AMD (Lutty et al., 2009; Bhutto and Lutty, 2012; Biesemeier et al., 2014; Mullins et al., 2014). Histologically, choriocapillaris tissue near the site of choroidal neovascular lesions exhibit decreased density without accompanying retinal pigmented epithelium disruption (Bhutto and Lutty 2012). Indeed, the choriocapillaris endothelium in aging macula is highly subject to complement activation stress and decreases in density with increasing drusen in dry or non-exudative AMD. Complement accumulation present in early stages may lead to choriocapillaris loss (Berenberg et al., 2012; Mullins et al., 2014). The resultant loss of vascular support to the retinal pigmented epithelium releases angiogenic signals which stimulate abnormal intravasation of choroidal vessels into subretinal layers, observed in some cases of nonexudative AMD which progress to wet AMD. Furthermore, it is well established that the functions of retinal pigmented epithelium and choriocapillaris show tight mutualistic dependence and atrophy of either structures leads to a dysfunction of the other (Blaauwgeers et al., 1999; Marneros et al., 2005; Biesemeier et al., 2014; Seddon et al., 2016; Chirco et al., 2017). Therefore, the pathogenesis of choroidal neovascularization may arise from initial structural changes in the vasculature (**Figure 1**).

### Vasculopathy in Choroidal Neovascular Lesions

In addition to vascular degeneration in the early stages of AMD, vascular dysfunction is manifested in late stage neovascular outcomes, such as polypoidal choroidal vasculopathy (PCV). PCV is a subtype of wet AMD that is most prevalent in Asians (Wong et al., 2014; Huang et al., 2016). It is characterized by abnormal branching vascular networks and a presence of polypoidal or aneurysmal dilations at the terminal ends of these networks. These polypoidal lesions appear as hyperfluorescent nodules in fundus indocyanine angiography (Yannuzzi et al., 1990; Liu et al., 2016). Choroidal vessels in PCV display hyalinization, an arteriosclerotic phenotype characterized by the replacement of smooth muscle tissue with ill-defined basement membrane-like material, as observed in histopathological sections (Leishman 1957; Okubo et al., 2002; Kuroiwa et al., 2004; Nakashizuka et al., 2008). The aneurysmal dilations observed at terminal ends of aberrant networks also have vascular causes. They have been purported to be a result of dysfunction of elastin, homocysteine-associated oxidative stress and endothelial dysfunction (Cheng et al., 2014). The presence of hyalinization and aneurysms clearly indicate that PCV is a vasculopathy of the inner choroidal vasculature with arteriosclerotic features.

Wong et al. (2016) have presented a comprehensive review of the epidemiology, detailed risk factors and clinical manifestations of two wet AMD subtypes - PCV and typical choroidal neovascularization. Mechanisms that could lead to common vessel wall pathology in PCV and typical choroidal neovascularization include impaired extracellular matrix metabolism (Nakashizuka et al., 2008; Jones et al., 2011), involvement of the high-density lipoprotein pathway (Liu et al., 2014), choroidal vascular hyperpermeability associated with genetic polymorphisms *ARMS2 A69S* (rs10490924) and *CFH* (rs1329428) (Yoneyama et al., 2016), and choroidal venous congestion leading to thickened choroid and choroidal vascular hyperpermeability in PCV (Chung et al., 2013) (**Figure 1**). Notably, levels of VEGF in the aqueous humor of patients with typical choroidal neovascularization were found to be higher than that in PCV patients. It is postulated that the two wet AMD subtypes could have different pathological mechanisms, with typical choroidal neovascularization being more VEGF-driven than PCV (Tong et al., 2006).

### Genetic Basis of Age-Related Macular Degeneration

In the past few decades, GWAS on AMD cohorts have revealed several disease-associated risk variants. The Genetics of AMD in Asians Consortium conducted a genome-wide and exome-wide association study to uncover the most common single nucleotide polymorphisms (SNPs) associated with wet AMD specifically in the East Asian population (Cheng et al., 2015). Consistent with previously identified variants, the SNPs *ARMS2-HTRA1* rs10490924, *CFH* rs10737680, *CETP* rs3764261, *ADAMTS9* rs6795735, *C2-CFB* rs429608, and *CFI* rs4698775 were the most significantly associated with wet AMD. In European and Asian populations, the most common SNPs seem to converge on the gene *HTRA1* and complement pathway-related genes. Advances in GWAS have generated more targets than biological interpretation can translate them into new therapeutics. Emerging functional studies are primarily focused on how those SNPs impact on retinal pigmented epithelium. We will further discuss how HTRA1 and complement factors could lead to aberrant vascular outcomes in a later section on *Vascular Mechanisms in Choroidal Neovascularization*.

The risk variant residing in proximity to the promoter region of *HTRA1* seems to be associated with elevated levels of HTRA1 in the retinal pigmented epithelium. It has been postulated that HTRA1 upregulation could lead to Bruch’s membrane matrix breakdown, hence promoting choroidal vessel invasion (Yang et al., 2006; Jones et al., 2011). Variants in complement genes *CFH*, *CFB*, *C2*, *C3*, *C5*, and *SERPING1* also suggest important roles of complement dysregulation in AMD (Khandhadia et al., 2012). SNPs affecting *CFH* and *C3* result in decreased CFH inhibition, thus leading to increased alternative complement pathway activation (Nishida et al., 2006; Yu et al., 2014; Zhang et al., 2018), which might contribute to an angiogenic microenvironment favoring progression to choroidal neovascularization. **Table 1** represents a brief overview on the topmost variants with known molecular effects and implicated mechanisms contributing to AMD. Genetics of AMD and PCV have been reviewed extensively by our colleagues (Wong et al., 2016).

**Table 1:** Common age-related macular degeneration (AMD) variants with known molecular effects and associated mechanisms.

Risk variant	Study references	Population/Type of cases	Effect of variant	Implicated mechanisms contributing to AMD
*ARMS2-HTRA1* rs10490924	(DeWan et al., 2006)	Asia (Hong Kong)/wet AMD	In linkage disequilibrium with rs11200638; surrogate marker for functional polymorphism rs11200638 (DeWan et al., 2006)	–
	(Fritsche et al., 2013)	Europe and Asia (Meta-analysis of GWAS)/advanced AMD		
	(Yu et al., 2011)	Europe (Meta-analysis of GWAS)/advanced AMD		
	(Cheng et al., 2015)	East Asia/wet AMD		
*HTRA1* rs11200638	(DeWan et al., 2006)	Asia (Hong Kong)/wet AMD	Increase in HTRA1 mRNA and protein [in RPE (DeWan, et al., 2006; Yang et al., 2006), in aqueous humor (Tosi et al., 2017) and in drusen (DeWan et al., 2006; Yang et al., 2006)]	Higher HTRA1 levels increase activity of degradative ECM enzymes and compromise Bruch membrane integrity, favoring choroidal invasion (Yang et al., 2006; Jones et al., 2011).
*CFH* rs10737680	(Fritsche et al., 2013)	Europe and Asia (Meta-analysis of GWAS)/advanced AMD	Loss of function mutation in CFH which disrupts binding of CFH to C3b *[Reported for common allele CFH Y402H and rare penetrant allele R1210C]* (Clark et al., 2010; Clark et al., 2013; Manuelian et al., 2003; Ferreira et al., 2009; Weismann et al., 2011)	Decreased CFH inhibition of C3b results in increased alternative complement pathway activation *[Reported for common allele CFH Y402H and rare penetrant allele R1210C]* (Clark et al., 2010; Clark et al., 2013; Manuelian et al., 2003; Ferreira et al., 2009; Weismann et al., 2011)
	(Cheng et al., 2015)	East Asia/wet AMD		
*C3* rs2230199	(Fritsche et al., 2013)	Europe and Asia (Meta-analysis of GWAS)/advanced AMD	Alteration of configuration of first ring of macroglobulin domains, reducing binding of C3 to CFH (protein studies using electron microscopy) (Nishida et al., 2006; Zhang et al., 2018)	Reduced C3 binding to CFH increases complement activation (Zhang et al., 2018)

Amongst the large repertoire of AMD single nucleotide polymorphisms (SNPs) generated by genome wide association studies (GWAS), several such as ARMS2-HTRA1 rs10490924, HTRA1 rs11200638, CFH rs10737680, and C3 rs2230199 have been further interrogated for their molecular effects and mechanisms leading to AMD.

### Limitation of Current Treatments

At present, gold standard therapy for wet AMD involves intravitreal administration of VEGF inhibitors such as bevacizumab, ranibizumab and aflibercept, based on the notion of VEGF being a main driver of angiogenesis (Xu and Chen, 2016; Siedlecki et al., 2017; Fogli et al., 2018). These are humanized monoclonal antibodies which act to decrease elevated VEGF at the site of neovascularization, eventually restoring retinal thickness and function (Golbaz et al., 2011). Other interventions for wet AMD include verteporfin photodynamic therapy, which is used in combination with anti-VEGF therapy to stimulate polyp regression in PCV (Cho et al., 2012; Qian et al., 2018). While anti-VEGF therapy has restored vision for many, the monotherapy does not improve visual acuity in a substantial number of AMD patients as a form of long-term management (Rofagha et al., 2013; Fernández-Robredo et al., 2014; Bracha et al., 2017; Dunn et al., 2017; Jaffe et al., 2017; Malek et al., 2018). Furthermore, approximately 15% of AMD patients do not respond to anti-VEGF treatment (Krebs et al., 2013). Zhang and colleagues have neatly reviewed potential mechanisms of resistance to anti-VEGF therapy (Zhang and Lai, 2018). In fact, the same anti-VEGF therapy tackling both PCV and typical wet AMD result in different treatment outcomes. PCV patients exhibit a poor response to anti-VEGF monotherapy compared to typical wet AMD patients (Gomi et al., 2008; Lai et al., 2008; Liu et al., 2016; Zhang and Lai 2018). While it reduced subretinal fluid, visual acuity and retinal thickness, anti-VEGF monotherapy failed to eliminate polypoidal lesions after a year of treatment, which could lead to recurrence of exudative maculopathy (Gomi et al., 2008; Lai et al., 2008; Tsujikawa et al., 2010). Considering these limitations of anti-VEGF therapy, there might be a need for alternative strategies targeting wet AMD upon greater elucidation of the mechanisms of wet AMD.

Photodynamic therapy has several limitations. Firstly, the procedure has considerable side effects. Photodynamic therapy could cause secondary subretinal hemorrhage, retinal pigmented epithelium tears, and choroidal ischemia, resulting in further visual deficit (Lai et al., 2004; Lee et al., 2008; Teo et al., 2018). Administration of photodynamic therapy may also exacerbate existing intraocular inflammation in PCV (Ho et al., 2016). Secondly, photodynamic therapy does not entirely occlude the branching vascular network in PCV eyes, allowing new active polyps to emerge from persistent networks and hence triggering disease recurrence (Akaza et al., 2007; Lee et al., 2008). Therefore, photodynamic therapy does not prevent recurrence of PCV. Lee and colleagues proposed that polyps are more susceptible to photodynamic therapy than the branching vascular network because verteporfin is mainly taken up by proliferative endothelial cells that express high low-density lipoprotein receptors. Endothelial cells at polyp sites are more proliferative than those at the branching vascular network and are therefore more susceptible to verteporfin. Overall, the presence of secondary inflammatory side effects and its inability to prevent recurrence have rendered photodynamic therapy questionable as an efficacious treatment option. The aforementioned issues contributing to current treatment limitations for wet AMD surface a key question of whether we have sufficiently understood the mechanistic underpinnings of exudative macular degeneration and PCV, and whether alternative therapeutic angles are possible.

## Linking Vascular Aspects From Ocular Disorders to Diseases of the Brain and Heart

Scientists and clinicians have traditionally viewed AMD as a stand-alone disease that is confined to the eye. However, recent results from large-scale epidemiological studies have consistently shown that AMD is associated with several other disorders (Cheung and Wong, 2014). It is important to note that due to AMD’s chronic degenerative nature, the disease tends to be associated with other chronic disorders such as cardiovascular and neurodegenerative disorders (Wong et al., 2006; Tan et al., 2008; Ohno-Matsui 2011; Lee et al., 2019). With accumulating evidence pointing to an increased risk of AMD in patients suffering from cardiovascular and neurodegenerative disorders and vice-versa, studies have started looking at common mechanisms that might underlie the associations. Here, we believe that blood vessels may provide some insights to the mechanistic link between AMD, cardiovascular, and neurodegenerative diseases.

### AMD and Dementia

With a globally aging population, age-related diseases such as AMD and dementia have received unprecedented attention. These diseases have been known to contribute largely to our economic burden and healthcare expenses (Gordois et al., 2012). A recent meta-analysis of association between the two diseases proved that they tend to comorbid (Rong et al., 2019). Furthermore, a longitudinal study which followed 3,877 dementia-free participants who were diagnosed with eye disorders, found that those with AMD had a 50% increased risk of developing Alzheimer’s disease later on (Lee et al., 2019). Interestingly, AMD and Alzheimer’s disease share several degenerative and pathological features such as oxidative stress, inflammation, and deposition of amyloid-rich materials (Beatty et al., 2000; Golde 2002; Lin and Beal 2006; Hollyfield et al., 2008). Such common pathological features between the two diseases may be attributed to the close anatomical link between the retina and brain, explained by their shared developmental origin from the neural tube. Recognized as “the window to the brain,” research have looked into using the blood vessels of the eye as a proxy to evaluate brain health (London et al., 2013; Lim et al., 2016; Yoon et al., 2019). While the vascular mechanisms that underlie the associations between AMD and dementia are largely still poorly described, here, we discuss two potential vascular links between AMD and dementia.

The first vascular link between AMD and dementia is highlighted by the deposition of vascular amyloid-β associated with tissue degeneration in both diseases. Traditionally, Alzheimer’s disease, the most frequent cause of dementia, is hypothesized to arise due to an imbalance between amyloid-β production and clearance, resulting in increased levels of amyloid-β in the central nervous system. Amyloid-β accumulation subsequently causes neurotoxicity and cognitive impairment (Hardy and Selkoe 2002). Similarly, deposition of amyloid-β at the site of choroidal vessels and in extracellular deposits known as drusen, has been found in AMD patients (Dutescu et al., 2009; Kam et al., 2010; Ohno-Matsui 2011; Wang et al., 2011). Multiple reservoirs of amyloid-β have been found in the aging retinas of AMD patients and elevated amyloid-β levels were found to be associated with the key stages of AMD progression (Ohno-Matsui 2011). The similarities in brain and ocular amyloid-β deposition suggest that similar pathogenic mechanisms might underlie these two diseases. From a vascular angle, amyloid-β has toxic effects on the vascular unit in both brain and eye. In cerebral amyloid angiopathy, a vascular abnormality frequently accompanying Alzheimer’s disease, amyloid-β directly hinders the adhesion of vascular smooth muscle cells to the basement membrane, leading to vascular damage (Mok et al., 2006). In the ageing retina, amyloid-β deposits from multiple reservoirs surrounding the retina exert pro-inflammatory and pro-angiogenic effects on the retinal pigmented epithelium, the choroidal vasculature and the neuroretina, which may lead to increased vascular permeability and triggering of choroidal neovascularization. This may occur on top of non-vascular effects of amyloid-β such as retinal pigmented epithelium degeneration and senescence and increased reactive oxygen species (Ratnayaka et al., 2015). Therefore, amyloid-β deposits may be a common mediator of vascular abnormalities in both AMD and Alzheimer’s disease.

With multiple failed clinical trials targeted at removing amyloid-β from the brain, researchers have turned to other possible hypotheses to explain the cause of Alzheimer’s disease (Holmes et al., 2008; Karran et al., 2011). The role of the blood vessel in cognitive dysfunction is well described by others (Snyder et al., 2015; Sweeney et al., 2018; Nortley et al., 2019). Cerebrovascular dysfunction might precede amyloid-β deposition in Alzheimer’s disease. In a 25-year longitudinal study on dementia, the presence of vascular risk factors at midlife was associated with higher levels of amyloid-β at late-life, indicating the role of vascular disease early in Alzheimer’s disease (Gottesman et al., 2017). Notably, adults with early cognitive dysfunction were found to develop brain microvascular damage independent of amyloid-β changes (Nation et al., 2019). Indeed, neuroimaging studies have found that patients with Alzheimer’s disease exhibit neurovascular impairment such as lowered cerebral blood flow and atherosclerotic vessels (Arvanitakis et al., 2016; van de Haar et al., 2016; Kisler et al., 2017). Postmortem interrogations of cerebral microvasculature depict reduced density, length, and diameter in Alzheimer’s disease compared with age-matched controls (Fischer et al., 1990; Buee et al., 1994; Bouras et al., 2006). These microvascular abnormalities show parallels to vascular dysfunction found in AMD. As described in an earlier section, such vascular dysfunction includes decreased choriocapillaris density in early stages of AMD and atherosclerotic features in choroidal vessels with polypoidal choroidal vasculopathy (Leishman 1957; Bhutto and Lutty 2012). Moreover, in Alzheimer’s disease, the breakdown of the blood-brain barrier is observed, often caused by cerebrovascular dysfunction (Sweeney et al., 2018; Nation et al., 2019). In AMD, the breakdown of the outer blood-retinal barrier is observed, due to cumulative pathological events affecting its key component – the retinal pigmented epithelium – and surrounding tissues involved – Bruch’s membrane and choroidal vasculature (Ambati, et al., 2013). These parallel pathologies may be explained by the functional and structural similarities of the blood-brain and blood-retinal barriers, both of which are derived from the developing neural tube (Ohno-Matsui 2011; London et al., 2013). Although we are still uncertain of the causative mechanisms that underlie AMD and Alzheimer’s disease, the similarity of vascular pathology between the two diseases highlights a possible mechanistic link between AMD and neurodegenerative diseases.

### AMD and Cardiovascular Diseases

Studies have found that changes in ocular microvascular pathology may be associated with underlying systemic vascular diseases such as cardiovascular disease. Increasing evidence has demonstrated that AMD may share identical risk factors and pathogenic mechanisms with cardiovascular diseases (Wu et al., 2014). In particular, both share several vascular-related factors highlighting the need to understand common mechanistic pathways that may result in an increased risk of developing one disease when one has the other (Tan et al., 2007; Yang et al., 2014; Pennington and DeAngelis 2016). For example, having atherosclerotic carotid arteries and hypertension may be linked to a higher risk of AMD (Vingerling et al., 1995; Cheung et al., 2007; Hogg et al., 2008; Klein et al., 2013). Conversely, AMD and atherosclerotic retinal vessels have been suggested to be a predictor of coronary artery disease (Tedeschi-Reiner et al., 2005; Thomas et al., 2015). Additionally, it has been proposed that inflammatory markers in the eye are linked with activation of inflammatory pathways in the heart (Seddon et al., 2004). Studies have uncovered the involvement of vascular-related molecular mechanisms such as chronic inflammation, endothelial dysfunction, and oxidative stress between AMD and cardiovascular diseases (Cai and Harrison 2000; Machalińska et al., 2012; Klein et al., 2014).

Endothelial dysfunction often refers to a range of deteriorative endothelial responses that includes altered vascular inflammatory responses, vascular growth dysregulation, and vascular remodeling impairments (Gimbrone, 1995). Clinical trials and research data have shown that endothelial dysfunction is implicated in AMD through dysregulation of VEGF and soluble ICAM1 secretion that is linked to neovascularization (Lip et al., 2001; Schaumberg et al., 2007). Similarly, studies have found that endothelial dysfunction precedes the development of atherosclerosis (Davignon and Ganz 2004; Mudau et al., 2012) and may be temporally associated with myocardial ischemia (Hasdai et al., 1997). Most recently, attention has been given to characterizing circulating endothelial cells as a hallmark of vascular impairments. Circulating endothelial cells, once part of the vascular endothelial monolayer, enter the bloodstream due to damage in the blood vessels. Notably, elevated number of circulating endothelial cells have been detected in individuals affected by cardiovascular diseases and AMD, which reflects vasculopathy in both diseases (Boos et al., 2006; Erdbruegger et al., 2006; Machalińska et al., 2011). Taken together, vascular-related injury is a common pathological pathway implicated in the pathogenesis of AMD and cardiovascular diseases.

Investigations into the molecular mechanisms that link similar pathologies observed in AMD, dementia, and cardiovascular diseases are still in its infancy due to limited understanding of the causative mechanisms of these diseases. We propose that a closer look at the vascular mechanisms could yield answers on the purported associations. Specifically, studies on vascular endothelial cells, smooth muscle cells and pericytes of the eye, brain, and heart can potentially illuminate pathways that connect these diseases.

## Experimental Models of Choroidal Neovascularization

As the causative mechanisms of AMD remain elusive, the use of experimental models that recapitulate clinical features accurately will greatly enhance our understanding of AMD etiology. Numerous *in vivo* and *in vitro* models have attempted to recapitulate the disease characteristics in its early and late stages. However, none have managed to recreate all the important pathological features seen in AMD owing to the disease’s complex interplay of genetic and environmental factors. This complexity is furthermore compounded by the differences in the ocular anatomy between animal models, cellular models and humans. Despite the limitations, existing animal and cellular models have uncovered important findings on the role of vascular system in wet AMD (Rosenfeld et al., 2006; Jager et al., 2008). As mentioned in our earlier section, current therapies aim at targeting blood vessel growth and angiogenic factors (Couch and Bakri 2011; Lally et al., 2012), with greater success of anti-VEGF therapy in certain subtypes of wet AMD. The focus has been pivoted toward the vascular system and the involvement of vascular-related molecular mechanisms in AMD pathogenesis. In this section, we review existing models of choroidal neovascularization and suggest potential improvements that could better enable the study of AMD pathophysiology.

### *In Vivo* Models

Animal models of choroidal neovascularization generally involve introducing a breach to the integrity of Bruch’s membrane in the macula. This is achieved using laser and light, surgical methods, or manipulation through transgenic animals. Out of the three methods, models of laser-induced choroidal neovascularization are most widely adopted (Lambert et al., 2013). The first *in vivo* model of choroidal neovascularization was developed by Ryan (1979) using photocoagulation methods to induce a defect in the Bruch’s membrane of the eyes of primates (Ryan, 1979). Building on this method, other groups were able to induce choroidal neovascularization with a higher rate of success in mouse models by modifying the different types of lasers (e.g., argon laser, krypton laser) and parameters targeted by the lasers (Dobi et al., 1989; Frank et al., 1989; Tobe et al., 1998).

The procedure to induce choroidal neovascularization in animal models starts with anesthetizing the animal and then dilating their pupils with an antimuscarinic drug, tropicamide. Laser photocoagulation is then performed to generate burns and laser spots in the areas of the eye surrounding the optic nerve. After laser treatment, the formation of a bubble at the burn spot indicates a rupture of the Bruch’s membrane and this is necessary for choroidal neovascularization to occur. Laser spots with bubbles would be continually observed posttreatment for the occurrence of choroidal neovascularization using imaging methods such as confocal microscopy (Kramer et al., 2000). Laser-induced choroidal neovascularization models have become a standard for treatment evaluation and studying *in vivo* mechanisms (Grossniklaus et al., 2010; Lambert et al., 2013). The merits of the model are that it is highly reproducible, inexpensive, and time-efficient to create. However, like the limitations of any *in vivo* model, the findings in animals may not be translated to humans. Compared to human eyes, mice and rats do not possess a macula in their eyes which proves to be a huge limitation when studying AMD as the main area of degeneration occurs at the macula. Furthermore, it is important to note that there are stark anatomical differences between biologically developed choroidal neovascularization and laser induced choroidal neovascularization in animal models. For example, undergoing the laser treatment could damage the neural retina, which is not typically affected in an individual with AMD, and these neuroretinal changes remove the biological similarity between experimental choroidal neovascularization and human choroidal neovascularization (Pennesi et al., 2012). In the end, it should be noted that laser induced acute injury does little to mimic the chronic onset of ocular neovascularization in diseases.

These laser-treated animal models have since been used to investigate the various molecular mechanisms of choroidal neovascularization and potential pharmacological interventions (Tobe et al., 1998; Bora et al., 2003; Tolentino et al., 2004; Jones et al., 2008). One of the more notable findings that led to current therapeutics was the importance of VEGF signaling in the development of choroidal neovascularization (Kwak et al., 2000). Treatments targeting VEGF signaling showed success in preventing vision loss and improving visual acuity for AMD patients at the early and late stages (Rosenfeld et al., 2006; CATT Research Group, 2011; Vogel et al., 2017). Apart from targeting VEGF, other studies open the possibility for therapeutics to inhibit and target other signaling pathways. Recently, apelin and TGF-β signaling were reported to play an essential role to trigger choroidal neovascularization in mouse models (Jiao et al., 2017; Wang et al., 2017). Additionally, transcriptional coactivator Yes-associated protein (YAP) was found to promote choroidal neovascularization formation by upregulating the proliferation of endothelial cells (Yan et al., 2018). Moreover, recent advances in nanotechnology have leveraged on choroidal neovascularization mouse models to pioneer a noninvasive method for treating choroidal neovascularization where local delivery of drugs are administered through light-triggered targeting (Wang et al., 2019). These findings underscore the value provided by *in-vivo* models of choroidal neovascularization.

Over the past few years, several optimizations and new developments have been made to augment existing *in vivo* models of laser-induced choroidal neovascularization (Poor et al., 2014; Gong et al., 2015). Recently, a preclinical mouse model of a complex heart disease was reported to accurately mimic the actual disease *in vivo* by combining the systematic manifestations of the disease instead of trying to recreate all the pathology (Schiattarella et al., 2019). In fact, AMD may be viewed as an manifestation of systematic disease (Cheung and Wong, 2014). Studies have widely reported associations between AMD and hypertension, cardiovascular disease, cerebrovascular disease, chronic kidney disease, and neurodegenerative disorders (Hogg et al., 2008; Nitsch et al., 2009; Kaarniranta et al., 2011; Chung et al., 2014). To the best of our knowledge, models of choroidal neovascularization created in combination with other stress paradigms such as metabolic perturbations have not been created. Perhaps scientists studying angiogenesis in ocular diseases such as AMD and diabetic retinopathy can apply similar principles to their animal models in order to account for systemic effects and interplay with other organ systems.

### *In Vitro* Models

With the recent announcement of the closing of Wellcome Sanger Institute animal research facility, it has signaled a shift in the scientific community’s preference for *in vitro* methods (Else 2019). Cellular systems are widely used as a working model for hypothesis testing due to their ease of handling, amenability to genetic manipulation and possibility to interrogate cell type-specific effects in isolation of other cofounding factors present in *in vivo* models. The use of primary choroidal endothelial cell lines has pinpointed signaling dysregulations in these cells as the main cause of new blood vessel formation in wet AMD (Wang and Hartnett 2016). However, human- and animal-derived choroidal endothelial cells can only be obtained post-mortem, making them a relatively scarce resource. Cells obtained from patients in advanced stage of AMD often limit their relevance in studying onset of choroidal neovascularization. Additionally, there are other constraints such as the difficulty in maintaining endothelial identity in long-term cell cultures (Rops et al., 2004).

The breakthrough by Takahashi and Yamanaka (2006) in discovering that differentiated cells can be reprogrammed back to its pluripotent state has revolutionized scientific research and allowed pluripotent stem cell derivatives to be used in place of primary cells (Takahashi and Yamanaka, 2006). Songstad et al. (2017) has reported success in generating choroidal endothelial cells from human pluripotent stem cells. They first reprogrammed human fibroblast from an individual with normal ocular history into induced pluripotent stem cells (iPSCs). These human iPSCs were differentiated alongside with a RF/6A cell line which was originally isolated from the choroid-retina of a rhesus macaque fetus (Lou and Hu, 1987). The differentiated choroidal endothelial cells expressed a choroid-restricted marker, carbonic anhydrase IV, and a fenestration marker. As part of the characterization, these differentiated cells were benchmarked against the transcriptomic signature of RF/6A cells (Songstad et al., 2017). However, a recent study led by Makin et al. (2018) conducted a rigorous characterization of the RF/6A cell line and found that RF/6A cells lack several key endothelial markers and phenotypic properties, hence limiting its use in validating iPSC-derived choroidal endothelial cells. It is still a common challenge in the iPSC field to achieve homogenous population of cell derivatives. Given that carbonic anhydrase IV is the closest and only known marker restricted to the choroid in the eye (Hageman et al., 1991), more research into specific cell fate markers would help in the generation of a pure population of these cells.

A recent study by Giacalone et al. (2019) discovered a way to immortalize human isolated choroidal endothelial cells by transducing them to express an endothelial cell specific promoter, CDH5p-hTERT/CDH5p-Tag. The immortalized choroidal endothelial cell line offers promise for a more reliable *in vitro* model as it expresses endothelial specific markers (vWF and CD34), the choroid-restricted marker carbonic anhydrase IV, AMD-related proteins (CFH), and display functional endothelial characteristics (Giacalone et al., 2019). On the other hand, scientists have cocultured choroidal endothelial cells with retinal pigmented epithelial cells to develop an *in vitro* disease model that more faithfully mimics the anatomical association of different cell types in the human eye [reviewed by Chichagova et al. (2018)]. As loss of functional cells occurs at the early stage of AMD, cell replacement therapy may potentially serve as a treatment for AMD (Veckeneer et al., 2017). Clinical trials for replacement with healthy retinal pigmented epithelial cells are underway. There remains concerns for possible complications such as the uncontrolled proliferation of lab-grown cells which have slowed down some of the trials (Garber 2015). Nevertheless, with more advanced technology, bioengineers are leveraging on 3D printing methods to create scaffolds of blood vessels for laboratory and clinical use (Huang and Zhang, 2014). Recently, a group led by Wimmer et al. (2019) successfully developed 3D blood vessels organoids that functioned strikingly similar to real human blood vessels when transplanted into mice (Wimmer et al., 2019). These *in vitro* vascular platforms offer a promising and exciting outlook for enabling research on vasculopathy as research continues to push the frontiers of creating functional human-like blood vessels.

Both *in vivo* and *in vitro* models of choroidal neovascularization have been developed for researchers to study pathology from molecular to cellular and system level. Each model has its own strengths and weaknesses. Both *in vivo* and *in vitro* models are complementary and can be manipulated appropriately to address certain limitations as well as develop more fit-for-purpose models of ocular angiogenesis.

## Vascular Mechanisms in Choroidal Neovascularization

**Figure 2:**
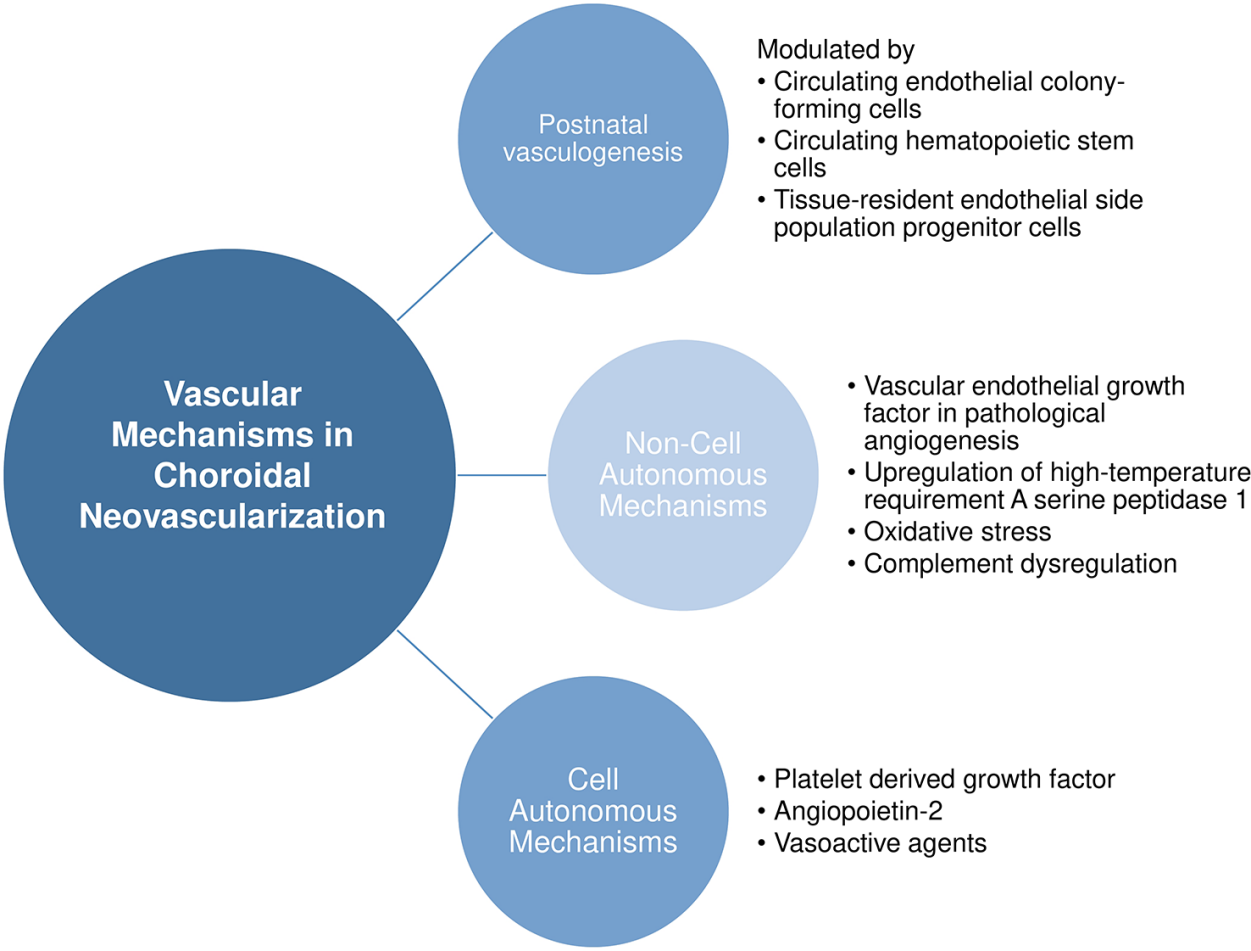
Vascular mechanisms in choroidal neovascularization.

Here, we provide a nonexhaustive review of the known vascular mechanisms in choroidal neovascularization, ranging from the source of pathological endothelial cells to noncell and cell autonomous mechanisms leading to choroidal neovascularization. **Figure 2** provides an overview of our discussion in this section.

### Source of Endothelial Cells in Choroidal Neovascularization

It was believed that all newly formed vessels in choroidal neovascularization arise from pre-existing choroidal vasculature (Ishibashi et al., 1987). However, in the 1990s, researchers discovered that circulating progenitor cells of bone marrow origin could in part contribute to postnatal vasculogenesis in both physiological and pathological neovascularization (Asahara et al., 1997; Asahara et al., 1999). Accumulating evidence then showed that circulating bone marrow progenitor cells contributed to newly generated endothelial cells specifically in choroidal neovascularization. Various groups have used the technique of transplanting EGFP-expressing bone marrow cells from EGFP donor mice into recipient mice and subsequently subjecting them to laser photocoagulation to induce injury in the choroid. The extent of donor derived GFP+ cells recruited to choroidal vasculatures or sites of Bruch membrane injury was then quantified. Often, GFP+ endothelial cells were found to give rise to different degrees of contribution to lesion endothelial cells (Sengupta et al., 2003; Tomita et al., 2004). Besides endothelial cells, a proportion of GFP+ cells was found to be immunoactive for vascular smooth muscle markers (Espinosa-Heidmann et al., 2003). Variability in levels of contribution to lesion endothelial cells was also observed depending on the stage of progression of choroidal neovascularization (Espinosa-Heidmann et al., 2005; Hou et al., 2006). In addition, circulating adult hematopoietic stem cells are mobilized into the injury region and are able to form endothelial cells that are subsequently incorporated into choroidal neovasculature (Chan-Ling et al., 2006). Such hemangioblast activity was also observed in a murine model of retinal neovascularization (Grant et al., 2002). Similarly, evidence of bone marrow contribution to choroidal neovascularization was observed in humans. Using AC133 a putative marker of both human hematopoietic stem and bone marrow-derived progenitor cells, Sheridan et al. identified the presence of bone marrow-derived progenitor cells in excised human choroidal neovascularization sections, albeit in very low numbers (Sheridan et al., 2006). **Table 2** presents a summary of the aforementioned *in vivo* studies.

**Table 2:** Summary of studies reporting bone marrow origin of endothelial cells in choroidal neovascularization.

Study references	Model species	Percentage of CD31+ endothelial cells in choroidal neovasculature that were bone-marrow derived	Total donor-derived bone marrow contribution to choroidal neovasculature	Percentage of bone marrow population in choroidal neovasculature that were endothelial
Tomita et al. ( 2004)	Murine	–	–	70%
Sengupta et al. (2003)	Murine	–	40 – 45%	–
Espinosa-Heidmann et al. (2003)	Murine	–	17%	41%
Takahashi et al. (2004)	Murine	5.3%	22% (total no. of cells: 154 ± 37; no. of marrow-derived cells: 34 ± 17)	20%
Espinosa-Heidmann et al. (2005)	Murine	65% in early choroidal neovascularization (3 days) 50% in late choroidal neovascularization (4 weeks)	20 – 40%	–
Hou et al. (2006)	Murine	70% in early choroidal neovascularization (7 days) 50% in late choroidal neovascularization (4 weeks)	–	31%
Sheridan et al. (2006)	Human	–	<0.1% stained for AC133	–

The mechanisms by which bone marrow progenitors are recruited to choroidal neovascularization sites have been described. Bone marrow derived cells incorporated into choroidal vasculature only at sites of laser-induced injury (Espinosa-Heidmann et al., 2003; Sengupta et al., 2003; Takahashi et al., 2004; Hou et al., 2006). This suggests that vascular injury is required for the mobilization of these cells and the microenvironment of the choroidal neovascular lesion might secrete molecular signals that assist in the recruitment and differentiation of circulating progenitor cells into vascular endothelial and smooth muscle cells *in situ* (Hou et al., 2006). Gao and colleagues have proposed that the process occurs in four phases: mobilization, migration, adhesion, and differentiation (Gao et al., 2016). Upon local tissue injury, the levels of various cytokines such as VEGF, granulocyte colony-stimulating factor (G-CSF), and erythropoietin (EPO) increase, which result in MMP9 activation, triggering the release of bone marrow cells from interacting stromal cells in the bone marrow (mobilization). The chemotactic gradients of cytokines then facilitate the migration of bone marrow cells to the local neovascular lesion site. Key chemokine mediators in choroidal neovascularization include the chemoattractant stromal derived factor (SDF-1), which is upregulated by retinal pigmented epithelium upon laser injury and binds to its concomitant receptor CXCR4 on bone marrow cells (Zhang et al., 2011). Cell adhesion molecules such as VCAM-1 and ICAM-1 then facilitate the adhesion of migrated bone marrow cells to existing endothelial cells at the site of choroidal neovascularization. The final phase of differentiation of the bone marrow progenitors to endothelial cells, smooth muscle cells, and macrophages then occur at the site of choroidal neovascularization. To summarize, choroidal neovascular injury specifically mobilizes and incorporates new vascular cells from the bone marrow into the injury site utilizing a complex repertoire of factors, pointing to the need to consider these processes in the pathological mechanisms of wet AMD.

**Figure 3:**
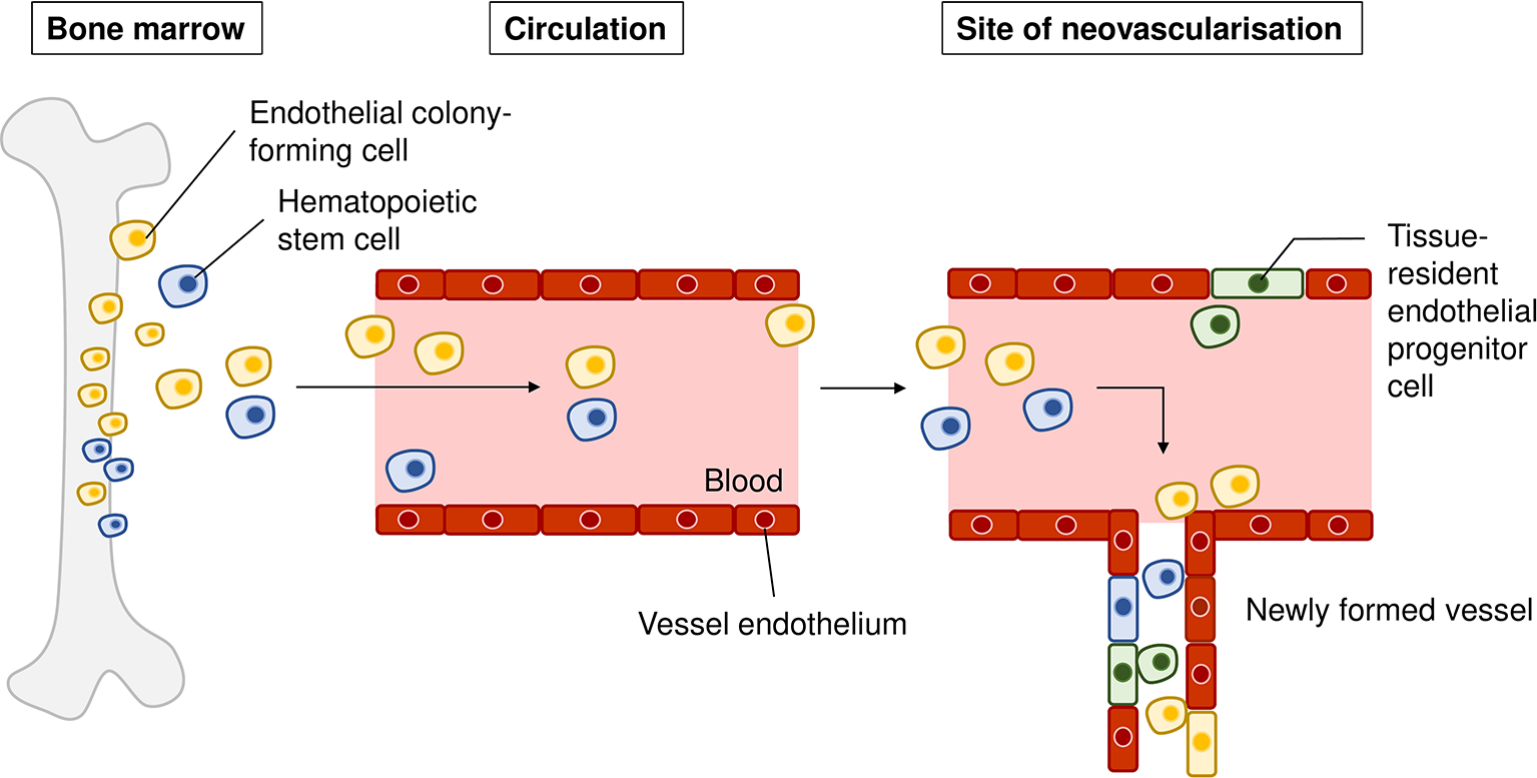
Sources of recruited endothelial cells in choroidal neovascularization. Endothelial colony-forming cells (yellow) from the bone marrow can be mobilized from the bone marrow into the circulation, migrate to the site of neovascularization, and differentiate into vascular cells that form the new vasculature. Hematopoietic stem cells (blue) can be mobilized to the site of injury, differentiate into endothelial cells and be incorporated into newly formed vasculature. Tissue-resident endothelial side population cells (green) residing in choroidal endothelium have been proposed to contribute to choroidal neovascularization upon injury.

Of note, there are also several studies that refute the contribution of bone marrow cells to postnatal vasculogenesis. Okuno and colleagues showed that bone marrow-derived cells did not contribute to the wound healing site as differentiated endothelial cells, but instead mainly as pro-angiogenic macrophages (Okuno et al., 2011). Grunwald et al. proposed that these recruited bone marrow cells are retained close to the neovasculature and exert proangiogenic effects on *in situ* endothelial cells (Grunewald et al., 2006). In line with the latter, Purhonen and colleagues demonstrated that during vasculogenesis none of the recruited bone marrow-derived cells contributed to the endothelium and contended that *in vivo* endothelial differentiation is a rare event for these cells (Purhonen et al., 2008). Alternatively, resident stem-like/progenitor cells have been discovered in pre-existing endothelium which demonstrate colony-forming ability (Naito et al., 2012). Wakabayashi and colleagues found that these resident progenitors (termed endothelial side population cells) did not originate from bone marrow and were thus distinct from bone marrow-derived endothelial progenitors. The endothelial side population cells isolated from murine choroidal tissue also displayed strong colony-forming ability *in vitro*, and increased proliferation upon laser-induced choroidal neovascularization *in vivo*, suggesting their ability to contribute to neovascular vessels (Wakabayashi et al., 2013). These studies highlight that postnatal vasculogenesis occurs to a significant extent in choroidal neovascularization. Taken together, endothelial cells that participate choroidal neovascularization could potentially originate from these sources: (1) circulating bone marrow progenitors, (2) circulating hematopoietic stem cells that have hemangioblast activity, and (3) vessel-residing endothelial side population cells that have high colony-forming activity. **Figure 3** provides a graphical representation of our discussion above.

### Noncell Autonomous Mechanisms

#### Vascular Endothelial Growth Factor in Pathological Angiogenesis

Dysregulation of VEGF signaling in lesion sites is known as one of the key stimuli for pathological angiogenesis (Kinnunen and Ylä-Herttuala, 2012). VEGF, existing as its various isoforms VEGF_121_, VEGF_145_, VEGF_165_, VEGF_189_, and VEGF_206_, is a potent angiogenic molecule that is known to stimulate proliferation, migration, tube formation, and vascular permeability of endothelial cells (Ferrara et al., 1991; Papadopoulos et al., 2012). The physiological importance of VEGF in the outer retina is well known. During fetal development, the retinal pigmented epithelium constitutively releases VEGF and FGF2 that are crucial for development of the choriocapillaris (Saint-Geniez and D’Amore, 2004; Anand-Apte and Hollyfield, 2010). VEGF released from the basal side of the RPE monolayer is required for the formation of fenestrations in the choriocapillaris (Blaauwgeers et al., 1999; Marneros et al., 2005). These important structures serve a role of allowing large macromolecules to be transported in and out of choroidal circulation (Anand-Apte and Hollyfield, 2010). *In vivo* studies report that knock-out of *vegf* in the RPE resulted in total ablation of the choriocapillaris (Korte et al., 1984; Kurihara et al., 2012). Therefore, locally synthesized VEGF from the RPE is critical for the maintenance of the choriocapillaris. In physiological conditions, ocular levels of VEGF are low, but in pathological conditions like choroidal neovascularization, VEGF levels are significantly elevated in affected sites (Kvanta et al., 1996; Kinnunen and Ylä-Herttuala, 2012). Of note, the VEGF isoform found to participate predominantly in pathological angiogenesis is VEGF_164/165_ (Ishida et al., 2003). In pathological angiogenesis, VEGF from hypoxic retina is believed to be the key driver (Miller et al., 1997; Papadopoulos et al., 2012). On top of its known functions of stimulating angiogenesis of choroidal vessels, elevated VEGF in the RPE leads to barrier integrity breakdown which could promote neovascularization (Ablonczy et al., 2011; Marneros 2013). As a proof of concept, treatment with VEGF antagonists have shown some success in reducing choroidal neovascularization lesion size and slowing the rate of vision loss (Schlingemann and Witmer, 2009; Kinnunen and Ylä-Herttuala, 2012; Papadopoulos et al., 2012). However, anti-VEGF drugs are not entirely effective to treat all choroidal neovascularization lesions in wet AMD and are also unable to prevent recurrence of symptoms, therefore pointing to the role of other interacting pathways of pathological neovascularization.

#### Upregulation of High-Temperature Requirement A Serine Peptidase 1

High-temperature requirement A serine peptidase 1 (HTRA1) is a multi-functional serine protease expressed in endothelium, epidermis, and neurons that regulates vascular growth and maintenance and is required for the normal development of vasculature in the brain and eye (De Luca et al., 2003; Jiang et al., 2012; Zhang et al., 2012). In 2006, it was reported that the SNP rs11200638 on the promoter sequence of *HTRA1* at chromosome 10q26 was the strongest casual genetic risk factor for AMD. The risk allele AA was associated with elevated levels of both *HTRA1* mRNA in lymphocytes of AMD donors and HTRA1 protein in retinal pigmented epithelium of AMD donors (DeWan et al., 2006; Yang et al., 2006). In line with these findings, Chan et al. found that HTRA1 mRNA expression was higher in the macula of AMD eyes with the AA genotype compared to non-AMD eyes (Chan et al., 2007). Elevated HTRA1 levels was also observed in the aqueous humor of patients with wet AMD (Tosi et al., 2017). The overexpression of human HTRA1 in the RPE of mice has been shown to result in development of PCV (choroidal lesions with polypoidal structure), although classic choroidal neovascularization formation was not observed (Jones et al., 2011). Furthermore, presence of HTRA1 protein was observed in the drusen of AMD patients (DeWan et al., 2006; Yang et al., 2006). Several mechanisms have been proposed for how HTRA1 overexpression could lead to choroidal neovascularization observed in wet AMD. Firstly, HTRA1 has been suggested to increase activity of degradative extracellular matrix enzymes and thus promote matrix breakdown (Grau et al., 2006; Jones et al., 2011). *In vivo* studies of transgenic mice overexpressing HTRA1 in the RPE described ultrastructural changes in Bruch’s membrane ECM (Vierkotten et al., 2011). It was therefore suggested that, in wet AMD, higher levels of HTRA1 compromise the integrity of Bruch’s membrane, allowing infiltration of choroidal vasculature through the layered matrix (Yang et al., 2006). Secondly, HTRA1 is also known to inhibit the activity of transforming growth factor (TGF-ß) family proteins which have important roles in angiogenesis and extracellular matrix production (Oka et al., 2004; Zhang et al., 2012). Mathura et al. reported that the TGF-ß proteins BMP-2 and BMP-4 might serve as repressors of RPE growth and any dysregulation of the BMPs might lead to aberrant wound repair as observed in proliferative retinopathies (Mathura et al., 2000). Therefore, increased levels of HTRA1 in the RPE of AMD patients with risk genotype might result in pathological choroidal neovascularization through (1) promoting degradation of Bruch’s membrane and compromising barrier function and (2) inhibiting BMP signaling thus removing a negative regulator for aberrant wound repair response.

#### Oxidative Stress

Antioxidants have been found to slow progression of progression from early AMD to advanced stages of AMD (wet AMD or severe geographic atrophy), thus highlighting the role of oxidative stress in AMD progression (Dong et al., 2009). Oxidative stress may facilitate in creating a pro-angiogenic environment in the outer retina and choroid, which coupled with altered integrity of Bruch’s membrane may trigger the development of choroidal neovascularization as observed in wet AMD (Dong et al., 2009). In addition, oxidative stress is also known to stimulate premature senescence of RPE, a key event in the pathogenesis of AMD (Supanji et al., 2013). Senescent RPE has been found to increase the expression of VEGF and downregulate CFH, both of which are known to contribute to the development of choroidal neovascularization (Marazita et al., 2016; Kaarniranta et al., 2018). Of note, Supanji et al. showed that oxidative stress stimulated RPE cells to increase production of HTRA1 which when in excess accelerated premature senescence of the RPE cells (Supanji et al., 2013), suggesting that HTRA1 also has a role in influencing RPE senescence. These studies point to the complex role of oxidative stress in contributing to ocular neovascularization.

#### Complement Dysregulation

The complement system participates in the innate immune response as the first immediate acting system before cellular response is carried out by macrophages and neutrophils. It is composed of more than 30 small proteins and activation products with chemotactic, inflammatory, cytotoxic, and antimicrobial functions (Zipfel, 2009). Once fully activated, formation of the membrane attack complex will occur, which is then be able to destroy cells and pathogens (Xu and Chen, 2016). Membrane attack complex deposition occurs naturally in healthy aging choriocapillaris (Mullins et al., 2014; Chirco et al., 2016). With a physiological balance of activation and repression of the complement system, self-tissue destruction is avoided. In AMD pathogenesis, a lack of repression of the complement system is implicated (Maugeri et al., 2018). Multiple complement products, such as C3, C5b-9, CFH, and CFB have been found in AMD lesions and drusen (Nozaki et al., 2006; Xu and Chen, 2016). Furthermore, as revealed by GWAS, genetic polymorphisms in complement genes such as CFH, CFB, C2, C3, C5, and SERPING1 confer risk for AMD, suggesting the role of complement dysregulation in the pathogenesis of AMD (Khandhadia et al., 2012). However, while numerous components are involved in AMD, only C3 and C5 have been reported for their roles in choroidal neovascularization. Nozaki et al. showed that induction of choroidal neovascularization *in vivo* increased levels of C3a and C5a, and C3a and C5a induced increase in VEGF secretion by primary human RPE *in vitro* (Nozaki et al., 2006). Knock out of the C3 gene protected mice from choroidal neovascularization after laser treatment (Bora et al., 2005), and genetic ablation of both C3a and C5a receptors results in lower VEGF secretion by RPE cells leading to decreased choroidal neovascularization (Nozaki et al., 2006). Overall, build-up of membrane attack complex in the choriocapillaris and dysregulated complement activation might contribute to an angiogenic environment for the development of choroidal neovascularization.

### Cell Autonomous Mechanisms

#### Platelet Derived Growth Factor

The long-term efficacy of anti-VEGF monotherapy on visual outcomes has been variable, with the need for repeated and lifelong treatment for patients with wet AMD (Singer et al., 2012; Rofagha et al., 2013; Silva et al., 2013). A common trend of initial visual improvement in the first few months followed by a plateau that lasts throughout the course of treatment has raised the notion of anti-VEGF resistance. In tumor studies, anti-VEGF resistance has been attributed to the secretion of platelet-derived growth factor (PDGF) by tumor cells which stimulate the recruitment and proliferation of pericytes to developing vasculature. On top of the physical stabilizing support rendered by pericytes, PDGF stimulates pericytes to upregulate VEGF which promote endothelial survival (Reinmuth et al., 2001; Franco et al., 2011). In choroidal neovascular sites, tip cells that form the vascular front express PDGF, causing recruitment of pericytes to the neovasculature and thereafter microvessel maturation. Newly recruited pericytes form a protective barrier around the newly formed endothelium in the face of anti-VEGF therapy, reducing the effect of VEGF inhibitors and explaining the plateau phase in long term anti-VEGF treatment (Franco et al., 2011; Pachydaki et al., 2012). In line with this hypothesis, choroidal neovascular lesions from patients who were unresponsive to anti-VEGF therapy, were also found to be well-formed and “consistently exhibit pericytes” (Pachydaki et al., 2012).

Considering these findings, PDGF inhibitors were proposed in combination therapy with current anti-VEGF monotherapy for wet AMD. E10030 (Fovista; Ophthotech, New York, NY) is a DNA aptamer against PDGF that was recently assessed for its efficacy in combination therapy with the anti-VEGF treatment ranibizumab (Lucentis). The results from a phase 2b clinical trial showed that there was a 62% benefit from baseline with combination therapy compared with anti-VEGF monotherapy (Jaffe et al., 2017). However, much to the disappointment of clinical investigators, the following two phase 3 trials showed that Fovista in combination with ranibizumab showed no superiority over ranibizumab monotherapy. Further on, two other phase 2 studies investigating Fovista in combination with two other anti-VEGF approved drugs were terminated (Dunn et al., 2017). The failed anti-PDGF clinical trials have taught us a few lessons: firstly, that phase 3 trials should not be designed based on retrospective subgroup analyses of phase 2 trial results (as was done in Fovista phase 3 trials) (Rosenfeld and Feuer, 2018), and secondly, that failure with PDGF antagonists indicate the need to shift efforts to target other mechanisms of choroidal neovascularization in AMD.

#### Angiopoietin-2

Angiopoietin-2 (ANG2) is a proangiogenic cytokine that plays a role in both angiogenesis and immune activation, both of which are integral processes in the pathogenesis of wet AMD (Fiedler et al., 2006; Wolf and Langmann, 2019). ANG2 levels have been found to be upregulated in aqueous humor of wet AMD human donors and increasing with disease severity (Ng et al., 2017). Due to its additional role in inflammation that is implicated in wet AMD, ANG2 has become a potential therapeutic target in wet AMD beyond anti-VEGF therapies (Gahn and Khanani, 2018). *In vivo* experiments have recently demonstrated ANG2 and VEGF combinatory inhibition led to reduced neovascular lesion formation in a spontaneous chronic choroidal neovascularization mouse model (JR5558 mice) (Foxton et al., 2019). This has been carried forward to phase 1 and 2 clinical trials with the bispecific antibody anti-VEGF-A/ANG2 (RG7116; Roche/Genetech). Currently, RG7116, now known as faricimab, is being tested in phase 3 trials in comparison with the VEGF trap drug aflibercept (Eylea) (Wolf and Langmann 2019). The shift in efforts towards VEGF-independent pathways in wet AMD is promising; and it is hoped that more novel targetable candidates would be uncovered.

#### Vasoactive Agents

Endothelial cells produce a physiological balance of vasoactive substances to regulate vascular function, such as the vasoconstrictor endothelin-1 (ET-1) and the vasodilator nitric oxide (NO). ET-1 levels increase while NO availability decreases during aging, resulting in increased vasoconstriction and impaired vasodilation, which could lead to constriction of smaller vessels associated with ischemia of the choriocapillaris (decreased choroidal blood flow) seen in severe dry AMD (Stauffer et al., 2008; El Assar De La Fuente et al., 2012; Totan et al., 2015). Decreased choroidal blood flow in dry AMD has been correlated with severity of dry AMD, and could increase the risk for ischemia and hypoxia leading to choroidal neovascularization in wet AMD (Grunwald et al., 2005). Totan et al. showed that patients with wet AMD exhibit increased ET-1 and decreased NO in the plasma, indicating that endothelial dysfunction is apparent in these patients (Totan et al., 2015). With age being the largest risk factor for AMD, it is not surprising that age-related vascular dysfunction would contribute to the progression of AMD, as seen also in a number of age-related diseases (Ehrlich et al., 2009; Akpek and Smith, 2013). Therefore, endothelial dysfunction in the choriocapillaris could play a role in AMD pathogenesis.

## Conclusion

With the great momentum in the study of choroidal neovascularization, there remains knowledge gaps which the scientific and clinical communities could address. We propose that further research on the following areas could be illuminating. (1) AMD subtypes could have different etiology, rendering it important to investigate subtype-specific mechanisms. Genomics distinguishing typical AMD and polypoidal choroidal vasculopathy may elucidate subtype-specific mechanisms. (2) Perturbations to choroidal vasculatures may have to be looked in the context of other influences. Existing experimental models could be adapted to recapitulate potential systemic/immune factors, as well as to study endothelial interplay with other cell types. (3) Finally, there is a need to explore both VEGF- and non-VEGF pathways to enhance the success of combinatorial treatment. We hope that vascular-targeting strategy will help advance therapy for early intervention.

## Author Contributions

CC provided the strategic focus for this review paper and edited the manuscript. NY and EC wrote and prepared the manuscript.

## Funding

We are grateful for the funding support of an Academic Research Fund (AcRF) Tier 2 grant (MOE2018-T2-1-042) from the Ministry of Education, Singapore, as well as support from the SERI-IMCB Program in Retinal Angiogenic Diseases (SIPRAD) grant number SPF2014/002, a grant from A-STAR (Agency for Science Technology and Research, Singapore).

## Conflict of Interest

The authors declare that the research was conducted in the absence of any commercial or financial relationships that could be construed as a potential conflict of interest.
